# Percutaneous retrieval via transseptal access of an Amplatzer Amulet left atrial appendage closure device embolized in the left ventricle

**DOI:** 10.1093/ehjcr/ytae656

**Published:** 2024-12-09

**Authors:** Alexandra Schratter, Jutta Benkö-Karner, Jutta Kunst-Hirnschall, Birgit Neunteufel, Shaojie Chen, Georg Delle Karth, Reinhard Achleitner

**Affiliations:** Department of Cardiology, Klinik Floridsdorf, Brünner Straße 68, 1210 Vienna, Austria; Department of Cardiology, Klinik Floridsdorf, Brünner Straße 68, 1210 Vienna, Austria; Department of Anaesthesiology, Klinik Floridsdorf, Brünner Straße 68, 1210 Vienna, Austria; Department of Cardiology, Klinik Floridsdorf, Brünner Straße 68, 1210 Vienna, Austria; Section of Cardiac Electrophysiology, Department of Internal Medicine B, University Medicine Greifswald, Fleischmannstraße 8, 17475 Greifswald, Germany; Department of Cardiology, Klinik Floridsdorf, Brünner Straße 68, 1210 Vienna, Austria; Department of Cardiology, Klinik Floridsdorf, Brünner Straße 68, 1210 Vienna, Austria

## Case report

An interventional left atrial appendage closure procedure was performed in an 87-year-old patient [general anaesthesia and transoesophageal echocardiography (TOE) guidance]. Landing zone was measured fluoroscopically according to the updated European Heart Rhythm Association/European Association of Percutaneous Cardiovascular Interventions expert consensus statement. A 28 mm Amplatzer™ Amulet™ left atrial appendage (LAA) occluder was implanted (Abbott, MN, USA). Fluoroscopic and TOE assessment showed satisfactory device shape and compression, and two tug tests confirmed a stable position inside the LAA.

The next day, X-ray and transthoracic echocardiography showed device embolization (*Panels A* and *B*, left; Supplementary material online, *Video S1*).

Re-catheterization was performed under TOE guidance (*Panel B*, right; Supplementary material online, *Videos S2* and *S3*). After transseptal puncture, a 12-French FlexCath Advance Steerable Sheath (Medtronic Inc., MN, USA) was positioned in the left atrium. An ablation catheter was introduced into the left ventricle, and the sheath was advanced beyond mitral annulus (*Panel C*) over the catheter, which was then retrieved and replaced by an MPA-1 catheter, with its tip pointing directly at the knob at the lobe side of the occluder. A multiplane flower-shaped snaring device (EN Snare 18–30 mm, 7 French, Merit Medical Systems Inc., UT, USA) was slowly pushed out of the MPA-1 catheter, engaging the knob of the occluder and then pulled back into the MPA-1 catheter, snaring the knob and fixing it to the tip of the MPA-1 catheter. By slow but continuous and strong pulling back of the snaring device, the occluder was pulled into the sheath (see Supplementary material online, *Video S4*) and retrieved without damaging the mitral valve (confirmed by TOE imaging).

The patient was discharged the next day.

**Figure:**
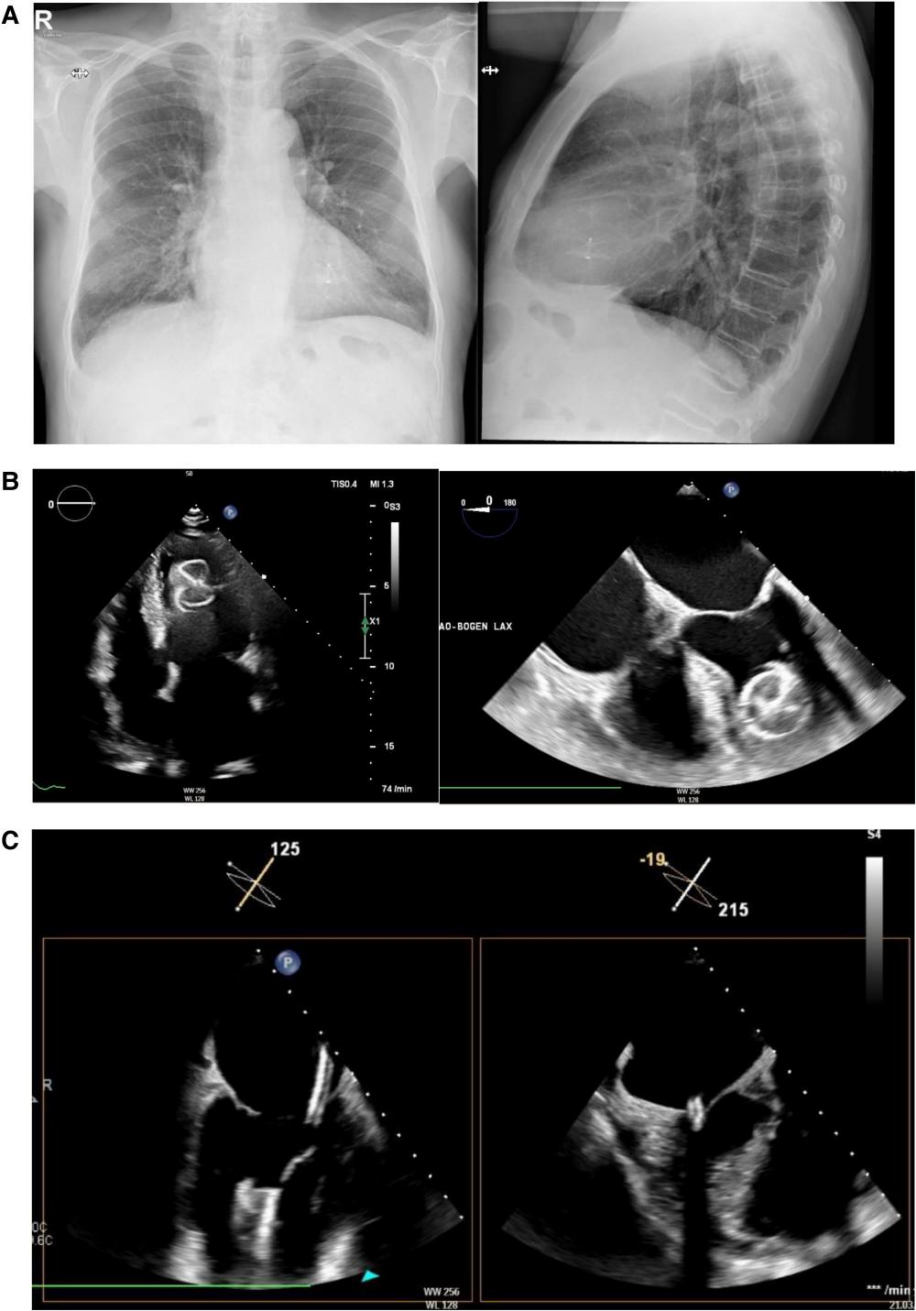


## Supplementary Material

ytae656_Supplementary_Data

## Data Availability

All available data are presented within the manuscript.

## Supplementary material

**Consent:** The patient gave written informed consent to the publication of this case.

## Notes

**Funding:** None declared.

